# Experimental Forelimb Allotransplantation in Canine Model

**DOI:** 10.1155/2016/1495710

**Published:** 2016-08-15

**Authors:** Sa-Hyeok Hong, Seok-Chan Eun

**Affiliations:** Department of Plastic and Reconstructive Surgery, Seoul National University College of Medicine, Seoul National University Bundang Hospital, Seoul 463-706, Republic of Korea

## Abstract

As reconstructive transplantation is gaining popularity as a viable alternative for upper limb amputees, it is becoming increasingly important for plastic surgeons to renew surgical skills and knowledge of this area. Forelimb allotransplantation research has been performed previously in rodent and swine models. However, preclinical canine forelimb allotransplantation studies are lacking in the literature. The purpose of this paper is to provide an overview of the surgical skills necessary to successfully perform forelimb transplantation in canines as a means to prepare for clinical application. A total of 18 transplantation operations on canines were performed. The recipient limb was shortened at the one-third proximal forearm level. The operation was performed in the following order: bones (two reconstructive plates), muscles and tendons (separately sutured), nerves (median, ulnar, and radial nerve), arteries (two), and veins (two). The total mean time of transplantation was 5 hours ± 30 minutes. All of the animals that received transplantation were treated with FK-506 (tacrolimus, 2 mg/kg) for 7 days after surgery. Most allografts survived with perfect viability without vascular problems during the early postoperative period. The canine forelimb allotransplantation model is well qualified to be a suitable training model for standard transplantation and future research work.

## 1. Introduction

Vascularized composite allotransplantation (VCA) is becoming a widely accepted alternative to upper limb amputation. Upper limb transplantation is also a prime example of VCA, comprising the subcutaneous, neurovascular, and mesenchymal tissues, such as the bone, cartilage, muscle, fascia, and skin [1]. In practice, they are faced with various medical, administrative, social, ethical, and regulatory challenges. Although many issues still require resolution, reconstructive surgeons should be prepared and consider VCA as the final option in reconstructive surgery. The outcomes of proximal forearm transplantation are hopeful [2, 3]. Before clinical application, however, the transplantation procedure requires thorough validation in animal models; thus, it is necessary to find a suitable animal model to further study correlative problems [4–6]. An ideal experimental model for upper limb transplantation should consider the following: feasible animal size, anatomic variance, vessel pedicle consistency, reasonable cost, operation time, ease of animal care, and so forth. Limb allotransplantation has been performed formerly in rodent [7, 8] and swine [9–11] models. There has previously been one study on limb allotransplantation using canines hind limbs [12]; however, canine forelimb allotransplantation has never been discussed in the literature. Developing an upper limb allograft model in canines is more appealing than other large animals, simply because the anatomy of canine forelimb—including upper extremity nerve and vascular anatomy—most closely resembles that of humans (aside from nonhuman primates). As such, using canines to develop an animal model for upper limb allotransplantation seemed most logical. The purpose of this paper is to provide an overview of surgical techniques involved in canine forelimb transplantation to be prepared for clinical application.

## 2. Materials and Methods

### 2.1. Experimental Animals

Two-year-old Beagles, weighing 12–15 kg, were used in this study. We closely followed the* Guide for the Care and Use of Laboratory Animals*, National Research Council. To ensure that transplants were exchanged between unrelated animals, their pedigrees were checked for at least two generations. Use of all animals in these experiments followed the guidelines for humane treatments of animals. A total of 18 transplantation operations were performed. All surgical procedures were performed under sterile conditions. Anesthesia was induced with ketamine and maintained with enflurane inhalation via tracheal intubation. During the procedure, animals were kept warm with a light source and a heating pad. The skin was cleansed with povidone-iodine (10%) solution and an antibiotic (potassium penicillin, 100,000 IU/kg, administered intramuscularly) was prophylactically administered before surgery. Lactated Ringer's solution was administered as a fluid supplement during and after the operation.

### 2.2. Preparation of the Donor and Recipient

**Figure 1:**
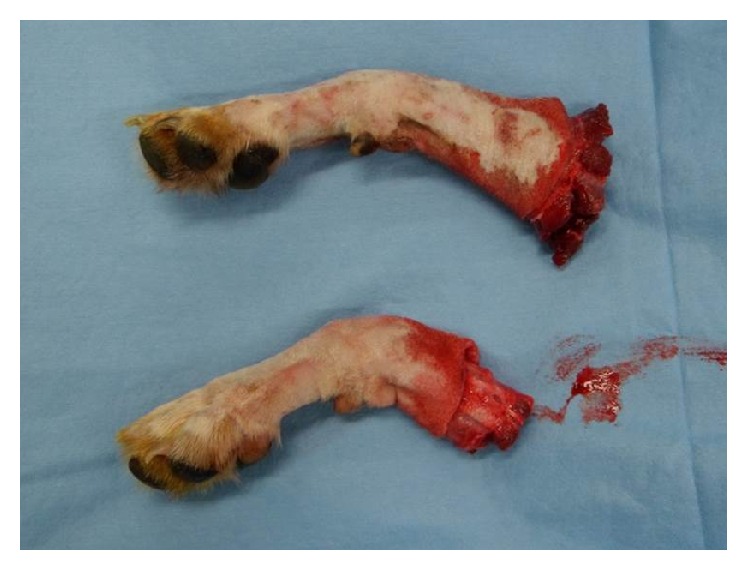
The radius and ulna are cut using an electrical saw at the midradius level.

Two teams performed the surgery simultaneously on both the donor and the recipient, attempting to shorten the operation time. On the donor's arm, a circumferential incision was made. The flexor and extensor muscle groups were elevated off of the ulna and the radius. The radius and ulna were cut using an electrical saw at the midradius level (Figure 1). The radial and ulnar arteries were arranged at the midforearm level for reanastomosis. The median nerve, ulnar nerve, and radial nerve were all prepared with as much length as possible.

Under tourniquet control, anterior and posterior skin flaps were elevated on the recipient's arms, and all neurovascular structures and muscles were identified and prepared. The medially and laterally originating muscles were dissected free from the ulna and radius. Then, the radius and ulna were cut using an electrical saw at the midradius level. The recipient's limb was shortened at proximal 1/3 level of the forearm.

### 2.3. Transplantation of the Vascularized Composite Allograft

**Figure 2:**
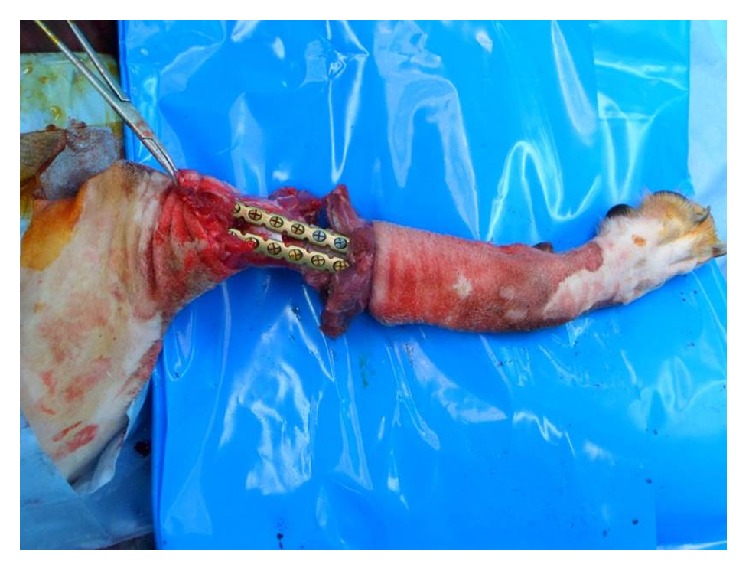
Bone fixation using two reconstruction titanium plates.

**Figure 3:**
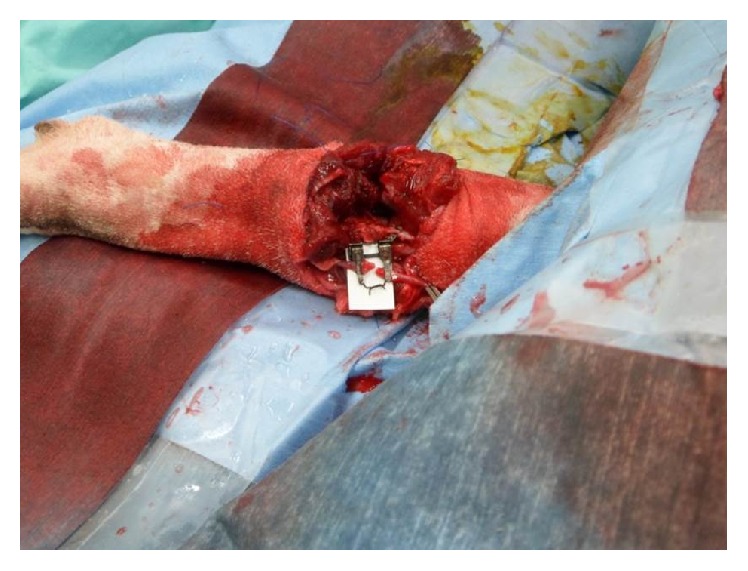
The attachment of three nerves (median, ulnar, and radial nerve) and the anastomosis of two arteries and two veins.

The sequence of transplantation then followed a standard replantation sequence with vascular repair that occurred immediately after bone fixation (osseous fixation, arterial repair, and venous repair, followed by nerve and muscular repair). Bone alignment ultimately dictates the pronation-supination range of motion in the proximal both-bone fractures, replantation and transplantation. Both plates were initially placed on the recipient's bones and then temporarily fixed to the donor. After bone fixation, the muscle groups were approximated using two reconstruction titanium plates (Figure 2). Attachment of the three nerves (median, ulnar, and radial nerves) and anastomosis of the two arteries and two veins were performed using nylon 9-0 under microscopy (Figure 3). The final skin suture marked the end of the transplantation surgery.

### 2.4. Treatments and Observation

**Figure 4:**
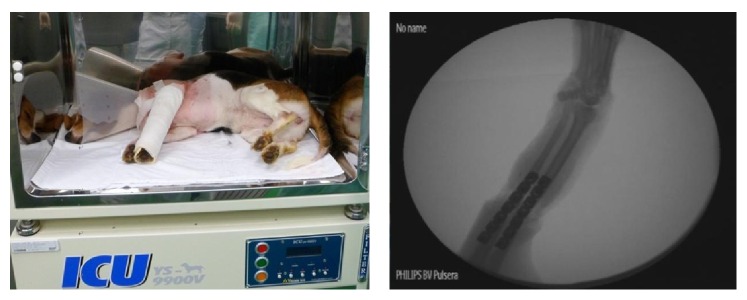
Postoperative course performed with immobilization at an intensive care unit (ICU).

The recipients received intravenous lactated Ringer's solution to compensate for perioperative fluid loss. They were immobilized and meticulously monitored throughout the postoperative course, with particular attention to adequate oral and fluid supplementation to facilitate recovery. A postoperative forelimb X-ray was taken at the intensive care unit (ICU) (Figure 4). All of the animals that received transplantation were treated with FK-506 (tacrolimus, 2 mg/kg) for 7 days after surgery. The transplanted upper limb flaps were evaluated every three hours for any clinical signs of rejection. Erythema, edema, loss of hair, desquamation, ulceration, and progressive shrinkage of the flap were considered to be clinical signs of rejection.

## 3. Results 

**Figure 5:**
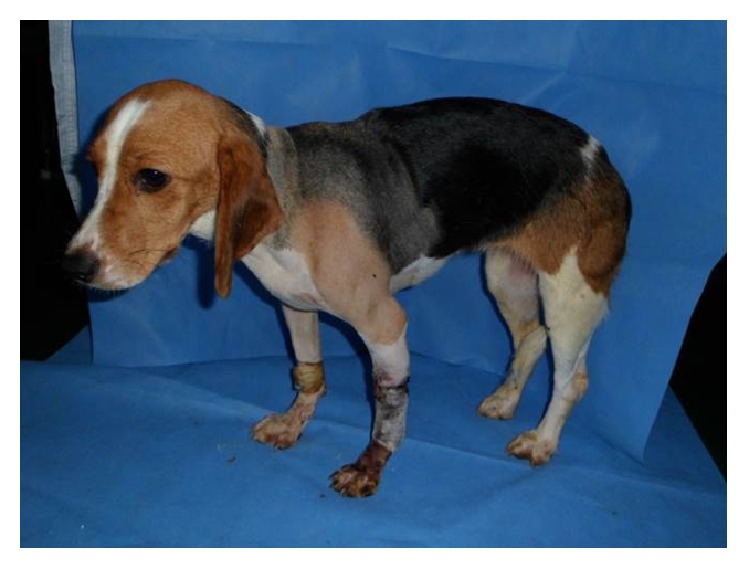
Posttransplantation appearance of forelimb allograft recipient at posttransplantation day 14.

**Figure 6:**
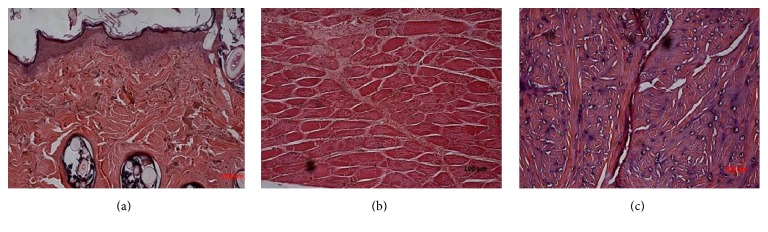
Biopsy samples taken from skin showed necrotic epidermis and edema typical of grade III rejection reaction (a), whereas biopsy specimens from the muscle showed grade II rejection of multifocal infiltration with myocyte necrosis (b) and cartilage showed grade I rejection of only focal erosion (c).

The mean time required to accomplish the upper limb transplantation procedure was 5 h and 30 min, and the mean time of warm ischemia was 45 min. A successful outcome was achieved by using the distal part of the radial and ulnar arteries as the recipient's arteries and the radial and ulnar veins as the recipient's veins. The mean diameter of the artery and vein was 4.0 mm (range: 3.8–4.2) and 6.2 mm (range: 5.8–6.4), respectively. On posttransplantation day 1, the animals returned to their normal routine of eating and drinking, but with immobilization. Mild soft tissue edema and hematomas under the flaps were observed, but no cases required drainage. One recipient showed acute rejection signs on posttransplantation day 5 and was sacrificed by intravenous administration of 100 mg/kg sodium pentobarbital on day 7. The remaining 17 recipients showed no signs of rejection until postoperative week 2 (Figure 5). The histologic outcomes were well correlated with the macroscopic appearance. The skin component showed signs of inflammation earlier and with greater intensity than other components, and it revealed epidermal edema and necrosis with massive neutrophilic infiltration in the dermis (grade III rejection reaction); the muscle and cartilage showed grades 2 and 1 rejection responses, respectively (Figure 6).

## 4. Discussion

Although there are only a limited number of reported forearm transplantation cases, they suggest favorable results [3]. Nonetheless, a highly scrutinized and well-established procedure from animal models is required before attempting upper limb allotransplantation on humans. For this purpose, we need to find the most suitable animal model to further our understanding of any potential correlative problems. In previous reports, rodent models have been attempted; however, they were too small and too different for establishing such models as the standard experiment for surgical skill training. In 1971, Lance et al. reported a canine hind limb model; however, nine dogs out of twenty lost the transplanted limb within the first postoperative week due to technical failures [12]. In our case, there was no transplanted limb loss due to technical failure.

There are many advantages to a canine forelimb transplantation model [13]. First, since canines are relatively larger animals with larger vessel size, compared with rodents, the operation, harvesting and insetting of the flaps, as well as microanastomosis, can be performed with relative ease. Second, the anatomical structure of canine forearms is comparable to that of humans. Third, the preoperative settings and anesthesia procedures are simple. Fourth, compared with other larger animals, the associated cost is lower, thus more cost-effective. Finally, the operation does not require advanced skills in microsurgery. As a result, a canine model can be implemented in many laboratories with minimal VCA experience.

Arterial and venous anastomosis is the most critical part of flap survival and overall success of the surgery. It is debatable whether to perform the flap inset or microanastomosis first [14]. Although the significance of early revascularization of the harvested flap cannot be underestimated, it is quite challenging to fix the flap safely without having the bone fixated using the titanium plates. Furthermore, the time required to fixate the bone was quite short in this model. Therefore, we performed microanastomosis after bone fixation. We have also performed nerve repair in this model, and, as aforementioned, due to the anatomical similarities between canines and humans, it is possible to practice surgical techniques before clinical application.

The animals were treated with low dose tacrolimus (2 mg/kg) only for 7 days, and the observation time was 2 weeks. Although this may be insufficient for the evaluation of long-term postoperative outcome, it is sufficient for evaluating the outcome of technical aspects of the operation, with respect to surgical techniques. If the operation was not successful, then there should be early failure in the transplanted limb within 2 weeks. Only 1 out of 18 recipients was sacrificed, and therefore we can safely assume that the outcome of the surgical procedures was excellent in this study. The primary purpose of this canine model was to simulate upper limb transplantation in animals best suited to prepare for clinical applications in humans. It was mainly intended to provide an overview of surgical simulation and techniques, and, as such, it was not focused on long-term postoperative function monitoring and failed to incorporate long-term immunosuppressive strategies to increase survival of the flaps. The effect of immunosuppressants in composite tissue transplantation is well documented in the literature [15–17]. A limitation to using a canine model is the lack of immune markers; there are a limited number of tools suitable for immunologic research—such as tolerance study—in a canine model compared with rodent models, such as mice or rats [13, 18, 19]. It is expected that further experimental investigations and future innovations might address the limitations posed by this model.

In sum, we have provided an overview of the canine forelimb allotransplantation technique and investigated its technical feasibility and applicability. The canine forelimb allotransplantation model is convenient, cost-effective, and reproducible. Moreover, it is suitable for training reconstructive surgeons who are not familiar with VCA procedures and lack advanced microsurgery skills. However, it is worthy to note that this model provides only a simplified version of flap harvesting and transferring procedures. The flap harvesting, transfer, and microscopic skills could be corrected in a better attainable method. Due to its feasible size and convenience for operation, canine forelimb allotransplantation model is a reasonable, reproducible, and representative upper limb transplantation study model.
